# An Efficient Transmission Power Control Scheme for Temperature Variation in Wireless Sensor Networks

**DOI:** 10.3390/s110303078

**Published:** 2011-03-10

**Authors:** Jungwook Lee, Kwangsue Chung

**Affiliations:** Department of Communication Engineering, Kwangwoon University, 447-1 Wolgye-Dong, Nowon-Ku, Seoul 139-701, Korea; E-Mail: jwlee@adams.kw.ac.kr

**Keywords:** wireless sensor networks, transmission power control, temperature, link quality

## Abstract

Wireless sensor networks collect data from several nodes dispersed at remote sites. Sensor nodes can be installed in harsh environments such as deserts, cities, and indoors, where the link quality changes considerably over time. Particularly, changes in transmission power may be caused by temperature, humidity, and other factors. In order to compensate for link quality changes, existing schemes detect the link quality changes between nodes and control transmission power through a series of feedback processes, but these approaches can cause heavy overhead with the additional control packets needed. In this paper, the change of the link quality according to temperature is examined through empirical experimentation. A new power control scheme combining both temperature-aware link quality compensation and a closed-loop feedback process to adapt to link quality changes is proposed. We prove that the proposed scheme effectively adapts the transmission power to the changing link quality with less control overhead and energy consumption.

## 1. Introduction

In low power wireless sensor networks, sensor nodes are widely deployed in various different environments to collect data. Because these nodes usually operate on limited battery power, energy efficiency is an important factor in protocol design. Each node communicates using a low power wireless link and its link quality varies significantly due to environmental dynamics. Packet loss is even more severe in harsh environments. Therefore, while maintaining good link quality with its neighbors, we need to reduce energy consumption for data transmission to extend the network lifetime.

Sensor nodes can be installed in a harsh environment in which temperature variation is serious. Since Received Signal Strength Indicator (RSSI) values tend to decrease when the temperature increases, connectivity between nodes can also be reduced [1]. For example, in the desert, the daily temperature range is extremely wide [2]. Similarly, in an urban area, the temperature variation is more serious due to the thermal island effect [3]. In a data center, servers generate heat and the link quality can be changed [4]. To compensate for temperature variation, temperature compensation devices are included as an enclosure for the AC power. Unfortunately, these devices can cause a large overhead because sensor nodes operate with batteries. Therefore, there is a need for new temperature compensation techniques.

Compared with the maximum transmission power, the controlled transmission power providing a fully connected network is more sensitive to temperature variation. It requires a more deliberate control mechanism to maintain link quality and causes inevitable control packet overhead. To efficiently compensate for the link quality changes due to temperature variations, in this paper we propose a new scheme for transmission power control that improves energy efficiency while achieving the required reliability. Our scheme aims to minimize control packet overhead for transmission power adjustment.

Our empirical experiments show that in real environments the temperature distribution is irregular and the link quality varies over time according to the temperature. A new scheme is proposed to combine on-demand open-loop and closed-loop feedback processes. In the open-loop feedback process, each node estimates the link quality using its temperature sensor. Estimated link quality degradation is then compensated by the transmission power control. In the closed-loop feedback process, the appropriate transmission power control is obtained by using additional control packets which are substantially less than those required in existing transmission power control schemes.

The remainder of this paper is organized as follows: In Section 2, we describe several existing transmission power control schemes. In Section 3, our empirical experiments on temperature variation are discussed. In Section 4, we describe our transmission power control scheme for temperature variation. Experimental results are presented in Section 5. Finally, we conclude in Section 6.

## 2. Related Works

To transmit data efficiently over wireless channels, existing schemes set some minimum transmission power for maintaining reliability. These schemes either decrease the interference among the nodes or the unnecessary energy consumption. In order to adjust the transmission power, a reference node periodically broadcasts a beacon message. When neighbor nodes hear a beacon message from a reference node, neighbor nodes transmit an ACK message. Through this interaction, a reference node can estimate the connectivity between the neighbor nodes.

In a Local Mean Algorithm (LMA) [5], a reference node broadcasts the “LifeMsg” message. The neighbor nodes transmit the “LifeAckMsg” after they receive “LifeMsg”. Reference nodes count the number of “LifeAckMsgs” and the transmission power is controlled by maintaining appropriate connectivity. For example, if the number of “LifeAckMsgs” is less than “NodeMinThresh,” the transmission power is increased. In contrast, if the number of “LifeAckMsgs” is more than “NodeMaxThresh,” the transmission power is decreased. As a result, they can provide improvement of network lifetime in a sufficiently connected network. However, LMA only guarantees connectivity between the nodes, but cannot estimate link quality [6–8]. Reliability eventually reduces due to the possibility of choosing a lossy link that affects irregular packet reception [9].

The Local Information No Topology/Local Information Link-state Topology (LINT/LILT) and Dynamic Transmission Power Control (DTPC) use the Received Signal Strength Indicator (RSSI). The nodes exceeding the RSSI threshold are regarded as the neighbor nodes with reliable links [10,11]. Transmission power can be controlled by a given Packet Reception Ratio (PRR) metric. Reducing the unnecessary control packet through blacklisting is also proposed [12]. While the PRR metric allows one to make more precise estimates, doing so requires several samplings, which decrease the agility of link quality estimation.

RSSI is inversely proportional to temperature and can differ by up to 8 dBm when the temperature changes from 25 °C to 65 °C. In other words, the RSSI threshold that satisfies the required PRR can change by up to 8 dBm. The Adaptive Transmission Power Control (ATPC) adjusts the transmission power dynamically according to spatial and temporal effects. This scheme tries to adapt the link quality that changes over time by using closed-loop feedback [7]. However, in large-scale wireless sensor networks, it is difficult to support scalability due to the serious overhead required to adjust the transmission power of each link.

Existing approaches estimate a variety of link quality indicators by periodically broadcasting a beacon. In addition, the feedback process is repeated for adaptively controlling transmission power. In adapting the link quality to the environments such as temperature variation, the packet overhead for transmission power control should be minimized. Reducing the number of control packets while maintaining reliability is an important technical issue.

In this paper, we propose a new transmission power control scheme to efficiently compensate for the changes of link quality according to the temperature. To reduce the packet overhead for power control, the temperature measured by sensors is utilized to adjust the transmission power level. By more accurately adjusting the transmission power, the closed-loop feedback process is additionally executed by using control packets.

## 3. Empirical Experiments

**Figure 1.**
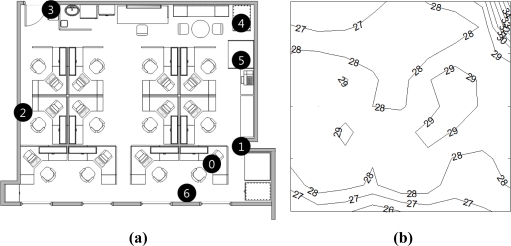
The layout and temperature distribution. **(a)** Layout; **(b)** Temperature distribution.

To analyze the change of link quality according to the temperature variation, we measured the RSSI in an indoor environment in which the temperature varied from 29 to 35 °C. Figure 1(a) shows the layout of the experimental environment. Our experiment is performed in an empty office to minimize effects on link quality variation from sources other than the temperature. We use TELOSB motes with CC2420 radio chips [13]. The TELOSB mote has a 12-bit resolution SHT11 temperature sensor and an integrated PCB antenna. In the experiment, the transmission power is set to 0 dBm that is the maximum value of CC2420. The packet rate is one packet per 5 seconds. Figure 1(b) shows the distribution of the daytime peak temperature in Figure 1(a).

**Figure 2.**
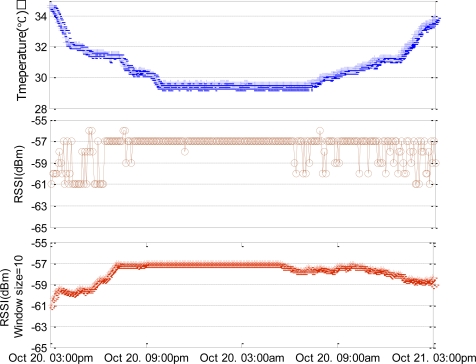
The change of RSSI according to temperature.

Temperature at Node 4 that is installed in the data sever is the highest. We measure the RSSI between Node 4 and Node B in the situation where the temperature changes over 24 hours. Figure 2 shows the RSSI corresponding to temperature variation at Node 4 (from 3 P.M. 20th October to 3 P.M. 21st October). The temperature variation is 6 °C over 24 hours. The RSSI becomes lowest when temperature is highest around 3 P.M. On the contrary, when a temperature is low, RSSI is high with less fluctuation. We can easily observe the inversely proportional relationship between RSSI and temperature. In a high temperature, the link quality is reduced and irregular.

**Figure 3.**
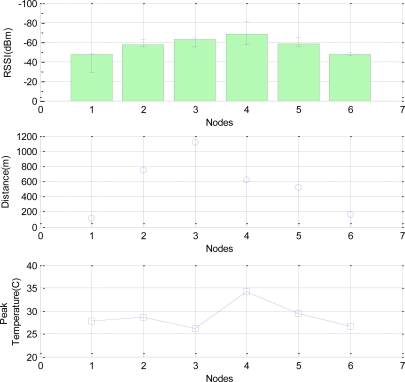
The correlation among distance, temperature, and RSSI.

As shown in Figure 3, we measured the RSSI between a sink node and six neighbor nodes and temperature at each node. Figure 3 shows that Node 4 reaches the highest temperature and has accordingly the lowest RSSI. Even though Nodes 2 and 3 are far away from the sink node, their RSSIs are higher than that of Node 4. By this experimental result, we confirm that the temperature is a very important factor to the RSSI value, that is, the link quality.

## 4. Proposed Transmission Power Control Scheme

In this section, we present a new transmission power control scheme that maintains the link quality during temperature variation. Our transmission power control scheme is designed to efficiently combine closed-loop and open-loop feedback processes. It utilizes the open-loop feedback process based on the sensed temperature information to reduce the overhead for the transmission power control according to temperature variation. The closed-loop feedback process based on control packets is further used to accurately adjust the transmission power. By adopting both open-loop and closed-loop feedback processes, we can achieve an efficient transmission power control for reliable links without excessive control packet overhead.

### 4.1. Combining the Open-Loop and Closed-Loop Feedback

**Figure 4.**
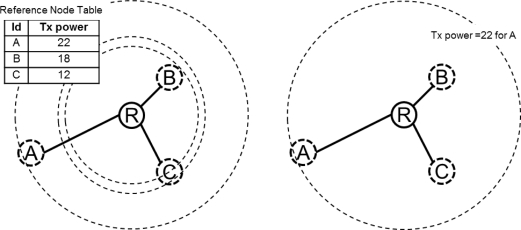
Link-level and network-level transmission power control.

Link-level control uses different transmission powers for different links, and network-level control chooses a single transmission power for all the links. It may be more energy efficient to control the transmission power for each link rather than to use network-level control. However, as shown in Figure 4, link-level control requires overheads to maintain the table for the transmission power of each link.

In order to assign the minimum and reachable transmission power to each link, the ATPC (Adaptive Transmission Power Control) is designed based on adjusting the transmission power for each link [7]. ATPC has two phases, *i.e.*, initial and run-time phases. In the initial phase, each node builds a model for each of its neighbors’ links. In the run-time phases, based on the previous model, ATPC adapts the link quality to dynamically maintain each link over time. In a relatively stable network, control overhead occurs only in measuring the link quality in the initial phase. In a relatively unstable network, because link quality is continuously changing, the initial phase is repeated and serious overhead can occur. In other words, if a node moves or the link quality is very irregular, adjusting the transmission power with network-level control is more efficient than link-level control.

Before we present the block diagram for the proposed scheme, several variables are defined as follows:

- Controlled number of neighbor nodes: *n_c_*(*t*)
- Desired number of neighbor nodes: *n_d_*(*t*)
- Error: *e*(*t*) = *n_d_*(*t*) − *n_c_*(*t*)
- Controlled transmission power: *txpow*(*t*)

**Figure 5.**
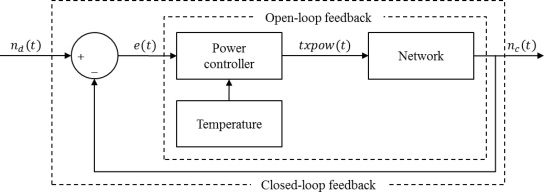
Block diagram for the proposed scheme.

In this paper, we propose a new transmission power control scheme based on network-level control. Figure 5 shows the system block diagram of the proposed scheme. In order to adjust the transmission power with network-level control, the transmission power level can be determined as connectivity with neighbor nodes. After comparing the number of current neighbor nodes with a set point (desired number of neighbor nodes), the controller adjusts the transmission power level accordingly. PRR (Packet Reception Ratio), ACK, and RSSI can be used to determine connectivity. ACK can estimate the connectivity, but it cannot determine the link quality. PRR can estimate the connectivity accurately, but it causes significant overhead due to many probe packets. In our scheme, we use the RSSI for connectivity estimation, which can measure the connectivity with relatively low overhead.

Power controller adjusts the transmission power level by utilizing both the number of current neighbor nodes and the temperature sensed at each neighbor node. Since our power controller is operated not merely by comparing the number of neighbor nodes with the desired number, but also by using the temperature-compensated power level, we can reach to the desired power level rapidly. If the temperature is changing, the temperature compensation is executed on the basis of the relation of a temperature and RSSI. The network connectivity can be maintained with low overhead by reducing the feedback process between nodes while the link quality is changing due to the temperature variation.

### 4.2. Temperature-Aware Transmission Power Compensation

The transmission power loss due to the temperature variation can be formulated using the relation between RSSI and the temperature experimented in Bannister *et al.* [1,14]. The equation for the RSSI loss for the temperature variation is as follows:

(1) $${\mathrm{RSSI}}_{\text{loss}}[\mathrm{dBm}]=0.1996\times (T[{}^{\circ}C]-25[{}^{\circ}C])$$

where T is the temperature in the range of 25 °C ≤ *T* ≤ 65 °C.

To compensate the RSSI loss calculated from Equation (1), we have to control the output power of TI CC2420 radio transceiver. The CC2420 is able to have 31 transmission power levels by setting the TXCTRL register appropriately. Table 1 shows the transmission power levels and their corresponding output power and current consumption of CC2420 according to the register values. Relations between power level and output power is formulated as Equation (2) by using a least square approximation:

(2) $$P_{\mathrm{level}}={\left\{\frac{P_{\mathrm{out}}[\mathrm{dBm}]+40}{12}\right\}}^{2.91}$$

where P_out_ is the output power of CC2420 and P_level_ is the corresponding power level.

Based on Equations (1,2), we can obtain the appropriate power level of the CC2420 to compensate the RSSI loss due to temperature variation by replacing P_out_ in Equation (2) by RSSI_loss_ in Equation (1). Our scheme aims to simplify the transmission power control by compensating the RSSI change based on the temperature information sensed at each node. The proposed compensation scheme does not require any communication overhead with neighbor nodes, but rather utilizes the information gathered from the temperature sensor located at a local node. This open-loop feedback control will reduce significantly the complexity of the closed-loop feedback control for transmission power control.

### 4.3. Details of the Proposed Scheme

We define important parameters: the RSSI threshold, the Max/Min number of the neighbor nodes and transmission power level as follows:

- RSSI threshold: RSSI_Th_ = −87 dBm
- Max/Min number of the neighbor nodes: Neighbor_max_ = 6, Neighbor_min_ = 5
- Transmission power control scale : 1 ≤ Δ ≤ 31

The RSSI threshold is the minimum value required to maintain the link reliability. Based on previous research [6], the PRR is at least 85% when the RSSI is above the threshold (−87 dBm). In this paper, we assume that the neighbor node with −87 dBm or greater of RSSI is reliable.

Max/Min numbers of the neighbor nodes are critical parameters for network performance. Kleinrock *et al.* [15] considered the problem of a set of nodes and tried to maximize the expected throughput that a packet can make toward its destination. They suggested that neighbor = 5.87 would indeed maximize throughput. We utilize this value and set Neighbor_max_ and Neighbor_min_ to 6 and 5 respectively.

**Figure 6.**
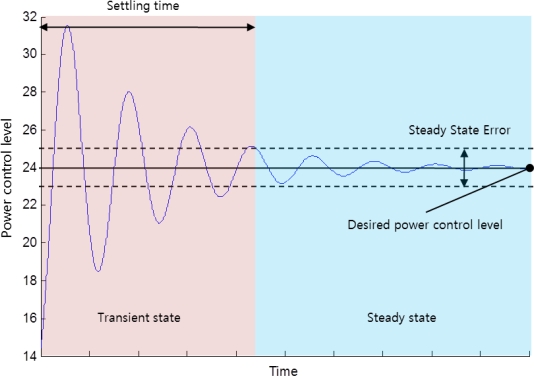
The transient state and steady state.

The transmission power control scale is also an important parameter to efficiently achieve the desired number of neighbor nodes by closed-loop feedback control. As shown in Figure 6, the transmission power control scale has a significant effect on settling time in which the transmission power reaches a steady state from the transient state. Before reaching to the steady state, many packet losses may occur. Longer transient state causes serious power consumption by repeatedly performing the closed-loop feedback process for transmission power control.

**Figure 7.**
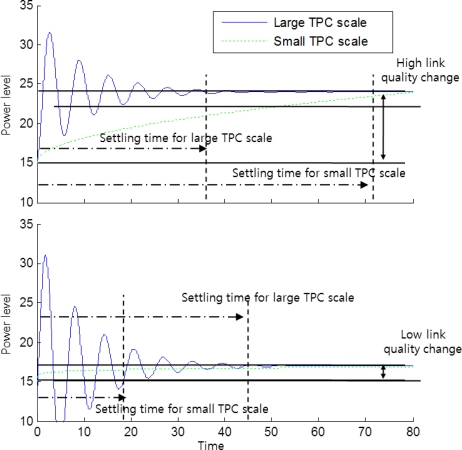
The correlation of the control scale and settling time.

As shown in Figure 7, the large transmission power control scale is more effective to reach a steady state rapidly when the link quality of a node changes drastically. On the contrary, if the link quality change is slow, the small transmission power control scale will be more efficient. Therefore, the transmission power control scale has to be determined considering the link quality changes. When the temperature changes, which causes the link quality changes, we compensate the temperature change by using Equation (2). We call this temperature-aware open-loop feedback control. With this open-loop compensation, the closed-loop feedback process uses the smallest transmission power control scale for the precise adjustment of transmission power level to obtain the desired number of neighbor nodes.

**Figure 8.**
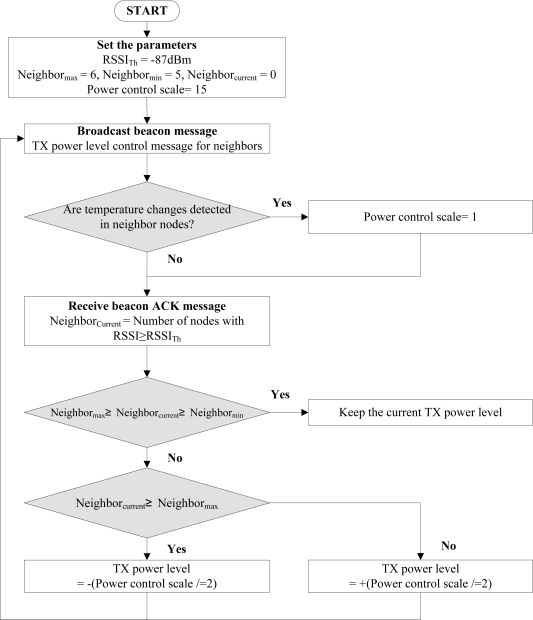
The flowchart of the reference nodes.

The flowchart for the reference node is shown in Figure 8. The reference node broadcasts the beacon message periodically to the neighbor nodes and waits ACKs. If ACKs are received from the neighbor nodes, RSSI for each node is measured and the number of nodes with sufficient RSSI value, RSSI > RSSI_th_, is counted. When the link quality change occurs due to factors other than temperature, such as the presence of an obstacle or movement of a node, the closed-loop feedback process controls the transmission power with an appropriate power control scale. For instance, in the case of the maximum transmission power level (31), the power control scales will change as 15, 8, 4, 2, and 1. It copes adaptively with the variation of the link quality. If temperature variation is detected from a neighbor node, the neighbor node compensates the transmission power by the open-loop feedback process. After the open-loop feedback process for temperature compensation, the power control scale is set to a minimum value, *i.e.*, 1, so that the closed-loop feedback process can control transmission power in a precise manner.

**Figure 9.**
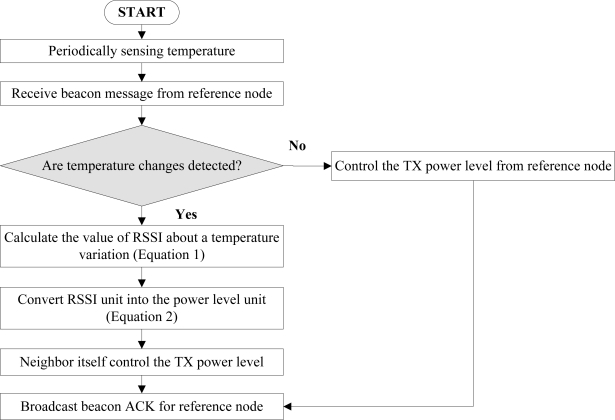
The flowchart of the neighbor nodes.

The operation of a neighbor node is described in Figure 9. Neighbor nodes receive the beacon message from the reference node. Then, the neighbor node senses the temperature by using locally installed sensor and checks if temperature change. If there is any temperature change, the compensation process is executed on the basis of Equations (1,2). The node sends an ACK message including the temperature change information with a newly calculated power level. Applying this temperature-aware compensation scheme first can reduce the overhead caused by the conventional closed-loop feedback control in changing temperature environments.

## 5. Experimental Results

**Figure 10.**
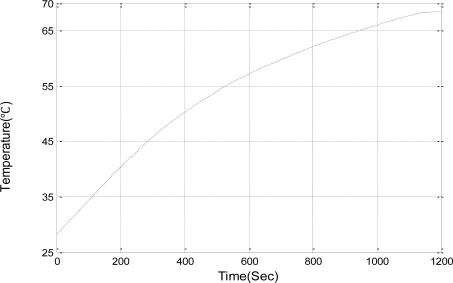
The temperature change.

For experiments, two TELOSB nodes are placed 7 meters away from each other and the transmission power used is 0 dBm. One node in the enclosure is exposed to a temperature variation from 28 °C and 68 °C caused by a heater. The enclosure has the maximum temperature, 68 °C, in our experiment. As shown in Figure 10, we increase the temperature from 28 °C to 68 °C over 1,200 seconds, in order to measure the RSSI variation.

**Figure 11.**
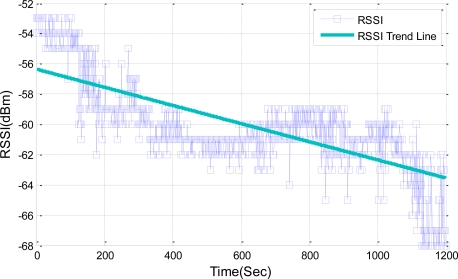
The measured RSSI according to the temperature change.

As shown in Figure 11, RSSI loss of the TELOSB mote is measured between 28 °C to 68 °C, with a maximum loss of 8 dBm at 68 °C. Increasing the temperature causes the RSSI loss over most of time. However, from 600 seconds to 900 seconds, the measured RSSI fluctuates and is a little increased while temperature increases. We guess this unexpected variation occurs due to factors other than the temperature. In the proposed scheme, these errors will be compensated through the closed-loop feedback control. The RSSI trend line in Figure 11 is approximately consistent with Equation (1).

To evaluate the performance, we compare our scheme with the maximum power, power level = 11 (PRR = 100% @25 °C), and DTPC in a changing temperature environment. Eight nodes are deployed, one of which is in the temperature changing enclosure. The maximum transmission power allows all the nodes to be connected. The power level = 11 maintains a maximum of six connections at 28 °C. DTPC and our scheme control the transmission power level every 1sec to maintain 5∼6 neighbor nodes.

**Figure 12.**
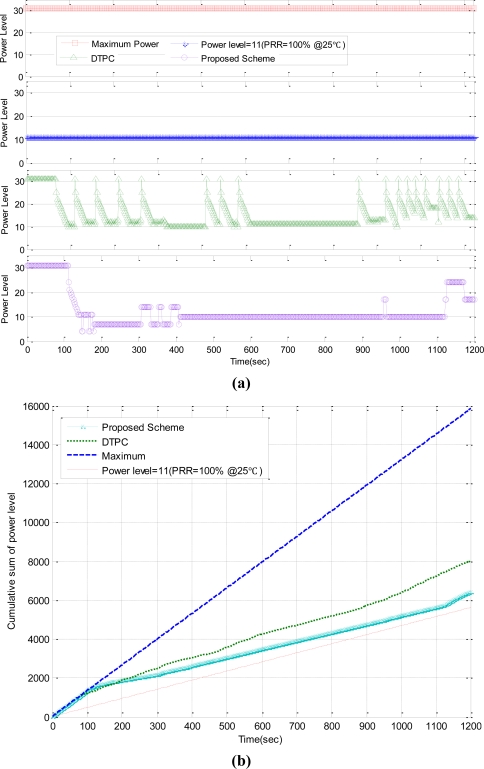
Energy efficiency measurement. **(a)** Power level, **(b)** Cumulative sum of power level.

While the temperature changes, the power levels are measured with various schemes, as shown in Figure 12(a). The cumulative sums of power levels are also shown in Figure 12(b). Power level = 11 has the lowest energy consumption, followed by the proposed scheme, DTPC, and the maximum transmission power (0 dBm). However, Power level = 11 causes many packet losses because it cannot adapt dynamically to the link quality changes.

**Figure 13.**
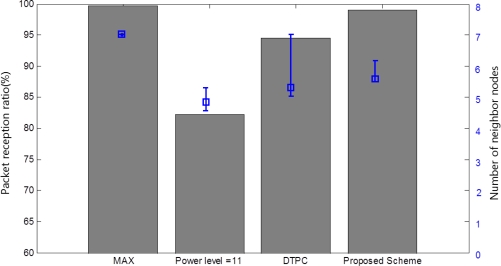
Packet reception ratio.

As shown in Figure 13, the maximum transmission power always maintains connectivity with seven neighbor nodes without adapting link quality changes. It provides almost 100% of the PRR. However, the maximum power leads the unnecessary energy consumption with its fixed maximum transmission power level as shown in Figure 12. Power level = 11 requires the minimum energy consumption. However, Power level = 11 has a poor PRR and fails to maintain the appropriate number of neighbor nodes, that is, 5 to 6, because it cannot provide the link quality adaptation according to temperature variation.

DTPC improves the reliability and energy efficiency through the transmission power control, comparing with fixed power schemes. However, since DTPC does not utilize the temperature sensed at each node and controls the transmission power merely by the closed-loop feedback method, the neighbor node connectivity is relatively unstable and its PRR is also lower than that of the proposed scheme, as shown in Figure 13. The proposed scheme and DTPC have 98.7% and 94.5% of PRR respectively. The proposed scheme generates less control packets than DTPC, because it controls the transmission power not only by the closed-loop feedback process, but also by temperature-aware compensation for link quality variation.

## 6. Conclusions

In this paper, we have presented an empirical study for the effect of temperature on wireless link quality. It shows that the temperature is one of most important factors impacting the link quality variation. The relationship between RSSI and temperature has been modeled for our transmission power control scheme. Our scheme uses open-loop feedback control to compensate for changes of link quality according to temperature variation. By combining both the open-loop temperature-aware compensation and the close-loop feedback control, we can significantly reduce the overhead of transmission power control in a wireless sensor network. In the future, we plan to further extend our scheme to consider other factors besides temperature affecting the link quality and to apply our scheme to a large-scale wireless sensor network.

## Figures and Tables

**Table 1.** CC2420 TXCTRL Register.

**Power Level**	**TXCTRL Register**	**Output Power**	**Current Consumption**
31	0xA0FF	0 dBm	17.4 mA
27	0xA0FB	−1 dBm	16.5 mA
23	0xA0F7	−3 dBm	15.2 mA
19	0xA0F3	−5 dBm	13.9 mA
15	0xA0EF	−7 dBm	12.5 mA
11	0xA0EB	−10 dBm	11.2 mA
7	0xA0E7	−15 dBm	9.9 mA
3	0xA0E3	−25 dBm	8.5 mA
